# Repeated neonatal sevoflurane induced neurocognitive impairment through NF-κB-mediated pyroptosis

**DOI:** 10.1186/s12974-021-02233-9

**Published:** 2021-08-21

**Authors:** Jing Dai, Xue Li, Cai Wang, Shuxin Gu, Lei Dai, Jingyun Zhang, Yunxia Fan, Jing Wu

**Affiliations:** 1 Department of Obstetrics and Gynecology, Affiliated Jintan Hospital of Jiangsu University, Changzhou, 213200 China; 2 Department of Anesthesiology, Affiliated Jintan Hospital of Jiangsu University, Changzhou, 213200 China; 3 Department of Anesthesiology, The First Affiliated Hospital of Zhengzhou University, Zhengzhou, 450052 China

**Keywords:** NF-κB, Pyroptosis, Neuroinflammation, Neurocognition, General anesthesia

## Abstract

**Background:**

Exposure to general anesthesia (GA) during the postnatal period is associated with neuroinflammation and long-term neurocognitive impairment in preclinical and clinical settings. Pyroptosis is a novel type of programmed cell death that, along with inflammation, has been found to play an important role in the mechanism of diverse neurological diseases. However, its roles in GA-induced neuroinflammation and neurocognitive impairment in the developing brain have not been investigated.

**Methods:**

Rats at postnatal day 6 or primary hippocampal neurons at 9 days in vitro received 3% sevoflurane for 2 h daily for three consecutive days. A pharmacological inhibitor of nuclear factor (NF)-κB (BAY 11-7082) was administered to suppress NF-κB activation. Histological and biochemical analyses were performed to assess the pyroptosis as well as neuronal and synaptic damage both in vivo and in vitro. In addition, behavioral tests were performed to evaluate neurocognitive ability in rats.

**Results:**

Repeated sevoflurane exposure activated NF-κB-mediated pyroptosis and neuroinflammation in the hippocampus in developing rats, damaged the neuronal morphology and synaptic integrity, and induced neurocognitive impairment in rats. BAY 11-7082 treatment suppressed the activation of pyroptosis, attenuated the neuronal and synaptic damage, and ameliorated the neurocognitive impairment induced by repeated sevoflurane administration to developing rats.

**Conclusions:**

Repeated sevoflurane GA may induce neuroinflammation and neurocognitive impairment in developing rats via the activation of NF-κB-mediated pyroptosis. Our findings characterize a novel role of pyroptosis as a potential therapeutic target in neuroinflammation after repeated neonatal GA.

## Introduction

Each year, increasing numbers of children and infants worldwide receive surgical and diagnostic procedures under general anesthesia (GA), often requiring repeated anesthetics exposure. Accumulating evidence from animal and preclinical studies has demonstrated that general anesthetics cause neuroinflammation in the developing brain, thus leading to neurodevelopmental deficits later in life [1–5]. Sevoflurane is the most commonly used inhalational general anesthetic in pediatric patients, because of its excellent respiratory tolerance, rapid onset, rapid offset, and hemodynamic stability [6]. Sevoflurane induces the activation of nuclear factor (NF)-κB, a master regulator of inflammation; in addition, it increases inflammatory cytokines, such as tumor necrosis factor-α (TNF-α), interleukin (IL)-1β and IL-6 in the developing brain, thus resulting in long-term cognitive dysfunction in adulthood [2]. Despite the role of NF-κB in neuroinflammation, a large gap remains in understanding of the consequences of NF-κB activation in the developing brain after neonatal general anesthetics exposure.

Pyroptosis is a novel inflammatory form of programmed cell death triggered by various pathological stimuli, such as stroke, cancer, and microbial infections [7–11]. This type of cell death is activated through the canonical nod-like receptor pyrin domain-containing 3 (NLRP3) inflammasome-caspase-1 pathway and non-canonical caspase-4/5/11 pathway. Specifically, activated inflammatory caspases cleave Gasdermin D (GSDMD) protein, the executor of pyroptosis, into two fragments (the N domain and C domain). Consequently, the N-terminal fragment of GSDMD (GSDMD-N) forms nanoscopic pores on the cell membrane, thus resulting in cell swelling and the release of proinflammatory molecules [7–11]. Our previous study has demonstrated that the canonical NLRP3 inflammasome-caspase-1 pyroptotic pathway is involved in cognitive impairment induced by the volatile anesthetic isoflurane in aged mice [12]. However, the links among general anesthetics, pyroptosis, and cognitive function remain largely unknown.

NF-κB is a nuclear transcription factor that participates in the control of a variety of cellular processes. Recent studies have demonstrated that activation of the NF-κB family of transcription factors is a key step in regulating pyroptosis, through promoting the transcription and translation of pyroptosis-related proteins including NLRP3, caspase-1, and caspase-11 [13–15]. Therefore, in the present study, we set out to investigate whether GSDMD-induced pyroptosis mediated by inflammatory caspases might be involved in the pathophysiology of neuroinflammation and cognitive deficits after repeated neonatal sevoflurane exposure in developing rats. In addition, BAY 11-7082, a selective NF-κB inhibitor [16, 17], was used to further investigate the link between sevoflurane GA and pyroptosis.

## Materials and methods

### Animals

Sprague-Dawley rat pups at postnatal day (PND) 6 were used in the present study. All experimental procedures and protocols were reviewed and approved by the Animal Investigation Ethics Committee of Jiangsu University and were performed in accordance with the Guidelines for the Care and Use of Laboratory Animals from the National Institutes of Health, USA. The pups were housed in a room maintained under constant environmental conditions (temperature 22–24 °C, a 12-h light/dark cycle, and 50 ± 10% humidity) with their mothers until PND 20. At PND 21, the pups were weaned and housed with four or five animals per cage under standard conditions.

### General anesthesia

Rat pups at PND 6 were randomly assigned to one of four treatment protocols: control + vehicle (Con group), control + BAY 11-7082 (Con + BAY group), sevoflurane + vehicle (Sev group), and sevoflurane + BAY 11-7082 (Sev + BAY group). BAY 11-7082 (Millipore Sigma, USA) was first dissolved in a small amount of DMSO and then diluted in phosphate-buffered saline (PBS) according to a previously published method [18, 19]. BAY 11-7082 (20 mg/kg) or PBS (vehicle) was intraperitoneally administered to the pups 30 min before gas inhalation [18, 19]. Sevoflurane anesthesia was induced by placing the rat pups in an anesthetizing chamber delivering 3% sevoflurane for 2 h daily for three consecutive days [20, 21]. In the control condition, 30% O_2_ was delivered at the same flow rate. The composition of the chamber gas was continuously monitored with a DatexTM infrared analyzer (Capnomac, Finland). Rats were kept at normothermic temperature throughout the experiment. Six rat pups from each group were sacrificed immediately after 2 h gas inhalation at PND 8, and their brains were rapidly removed for histological and biochemical studies. Twelve rats from each group were used for behavioral studies at PND 40, 50, and 60.

### Rat hippocampal neuronal culture and anesthetic exposure

Primary hippocampal neuronal cultures were prepared from embryonic day 16–17 Sprague-Dawley rat embryos, as previously described [22]. Neurons were dissociated and seeded on poly-D-lysine-coated plates or coverslips with neurobasal medium (Thermo Fisher Scientific, Waltham, MA, USA) supplemented with B27 (Thermo Fisher Scientific, USA), GlutaMAX-I (Thermo Fisher Scientific, USA), 5% FBS (Invitrogen GIBCO Life Technologies, USA), and antibiotics. After 2 h incubation, primary cultures were maintained in neurobasal medium without FBS in a 5% CO_2_ incubator at 37 °C. Subsequently, half the medium was replaced every 2 days.

After 9 days in vitro (DIV), the neurons received 3% sevoflurane or control gas (21% O_2_, 5% CO_2_, and 70% nitrous oxide) exposure for 2 h daily for three consecutive days at 37 °C. BAY 11-7082 (5 μM) or an equal volume of DMSO was added to the culture medium 30 min before GA exposure according to the conditions for each group [23].

### Cell viability assays

At DIV 11, neuronal cell viability was detected with a Cell Counting Kit-8 (Beyotime Institute of Biotechnology, China) according to the manufacturer’s instructions. Results are expressed as the percentage decrease in absorbance at 450 nm with normalization to the absorbance of the control cells.

### Measurement of mRNA levels

The total RNA in the rat hippocampi was extracted with TRIzol Reagent (Life Technologies, Inc., Grand Island, NY, USA). A First Strand cDNA Synthesis Kit (Thermo Fisher Scientific, USA) was used to synthesize cDNA. Real time-PCR was performed with SYBR Green PCR Master Mix (Thermo Fisher Scientific, USA). The sequences of the primers were as follows: NLRP3, forward 5′-CGGTGACCTTGTGTGTGCTT-3′ and reverse 5′-TCATGTCCTGAGCCATGGAAG-3′; caspase-1, forward 5′-GAACAAAGAAGGTGGCGCAT-3′ and reverse 5′-AGACGTGTACGAGTGGGTGT-3′; caspase-11, forward 5′-ATGTGGAGAAGGACTTCATTGC-3′ and reverse 5′-AGATGACAAGAGCAGGCATGTA-3′; and β-actin, forward 5′-TCAGCAAGCAGGAGTACGATG-3′ and reverse 5′-GTGTAAAACGCAGCTCAGTAACA-3′. The expression of β-actin was used as the internal control to assess the expression of target genes.

### Western blotting analysis

Protein quantification was performed with a Pierce BCA Protein Assay Kit (Beyotime Institute of Biotechnology), and 30–50 μg of total protein was resolved by polyacrylamide gel electrophoresis (SDS-PAGE, 8%). Protein levels were determined via incubation with antibodies against NF-κB-p65 (1:500; Abcam, UK), IκBα (1:500; Abcam, UK), NLRP3 (1:500; Abcam, UK), caspase-1 (1:500; Santa Cruz Biotechnology, USA), caspase-11 (1:500; Santa Cruz Biotechnology, USA), IL-1β (1:500; Santa Cruz Biotechnology, USA), IL-18 (1:500; Abcam, UK), Synapsin-1 (1:500; Millipore, USA), PSD-95 (1:500; Abcam, UK), GSDMD (1:500; Santa Cruz Biotechnology, USA), Lamin B (1:1000; Proteintech, USA), and GAPDH (1:500; Abcam, UK). The blots were imaged with ECL Plus western blotting detection reagents. ImageJ software was used to determine the average absorbance value of the corresponding bands.

### Enzyme-linked immunosorbent assay (ELISA)

The concentrations of IL-1β and IL-18 in the hippocampus were determined with an ELISA kit according to the manufacturer’s instructions (Abcam, UK). Briefly, the supernatants of hippocampal tissue were added to 96-well plates coated with the indicated antibodies. After the reaction between the enzyme and substrate, the absorbance values of the samples were assessed at 450 nm with a microplate reader (Thermo Fisher Scientific, USA).

### Immunocytochemistry staining

Immunofluorescence staining was performed as previously described. Briefly, neuronal coverslips were fixed with 4% PFA for 10 min and then permeabilized with blocking buffer comprising 5% goat serum, 1% bovine serum albumin, and 0.3% Triton X-100 at room temperature for 1 h. The samples were incubated overnight with primary antibodies against GSDMD (1:200, Santa Cruz Biotechnology, USA) and microtubule-associated protein-2 (MAP2) (1:500, Millipore Sigma, USA) at 4 °C, then incubated with the appropriate Alexa Fluor-488/594-conjugated secondary antibodies (Jackson ImmunoResearch, USA) and DAPI. Images were obtained through confocal microscopy (Fluoview FV 10i, Olympus, USA) and analyzed in FV10-ASW 2.1 Viewer software.

### Immunohistochemical staining

Immunohistochemistry was used to detect the immunoreactivity of GSDMD. The brain tissues were immediately perfused with 4% paraformaldehyde in PBS after removal and embedded in paraffin for sectioning. Brain sections (4 μm thickness) were incubated overnight at 4 °C with primary antibody against GSDMD (1:200, Santa Cruz Biotechnology, USA). The sections were then incubated with a secondary antibody labeled with horseradish peroxidase for 30 min at room temperature. Cells with brownish-yellow cytoplasm were counted as positive cells. For quantitative immunostaining, GSDMD-positive cells were observed under an inverted microscope, and the CA1 and dentate gyrus (DG) regions were counted for all groups in ImageJ software.

### Open field tests

At PND 40, each rat (*n* = 12 for each group) was gently placed in the center of a black plastic chamber (100 cm × 100 cm × 40 cm) for 5 min. The exploratory behavior was automatically recorded by a video tracking system (XR-XZ301, Shanghai Xinruan Information Technology Co., Ltd., China). The total distance and the amount of time traveled in the center area (50 cm × 50 cm) of the maze were measured. After each test, the arena was cleaned with 75% alcohol to avoid the presence of olfactory cues.

### Morris water maze (MWM) tests

MWM tests (XR-XM101; Shanghai Xinruan Information Technology Co., Ltd., China) were performed at PND 50. In the training phase, each rat was allowed to face to the pool wall in four random places (north, south, east, or west) in the pool to find the fixed platform. The trial was terminated after the rat reached the platform. If the rat did not reach the platform within 60 s, it was guided to the platform and allowed to stay for 10 s, and then the latency was recorded for 60 s. In the probe test, single-probe trial was conducted with the original platform removed 24 h after the last training session. The rat was released at the opposite position of the platform and allowed to swim for 60 s in the pool.

### Fear conditioning tests

Fear conditioning tests (XR-XC404; Shanghai Xinruan Information Technology Co., Ltd., China) were performed at PND 60. Each rat was placed in a conditioning chamber and allowed to explore freely for 3 min. Then, one tone-foot-shock pairing (tone, 30 s, 85 dB, 2 kHz; foot-shock, 2 s, 0.8 mA) was delivered. The rat then remained in the chamber for another 30 s and was subsequently returned to the home cage. The contextual fear conditioning test (a hippocampus-dependent task) was performed 24 h later by placing each rat back in the same test chamber for 5 min without any stimulation. Two hours later, the tone fear conditioning test (a hippocampus-independent task) was performed by placing each rat in a novel chamber with a different shape, color, and smell from the previous chamber, and the same tone was presented for 3 min without foot shock. Freezing behavior, defined as the absence of all visible movement except for respiration, was automatically recorded by the video tracking system.

### Statistical analysis

Data are presented as the mean ± SEM and were analyzed in GraphPad Prism 8.0 software. The differences between groups were determined with one-way analysis of variance followed by Tukey’s test. Comparisons for the spatial training sessions of MWM were performed with repeated two-way ANOVA followed by LSD test. A *p* value < 0.05 was regarded as indicating statistical significance.

## Results

### Repeated sevoflurane administration activates NF-κB signaling in the hippocampus in neonatal rats

**Fig. 1:**
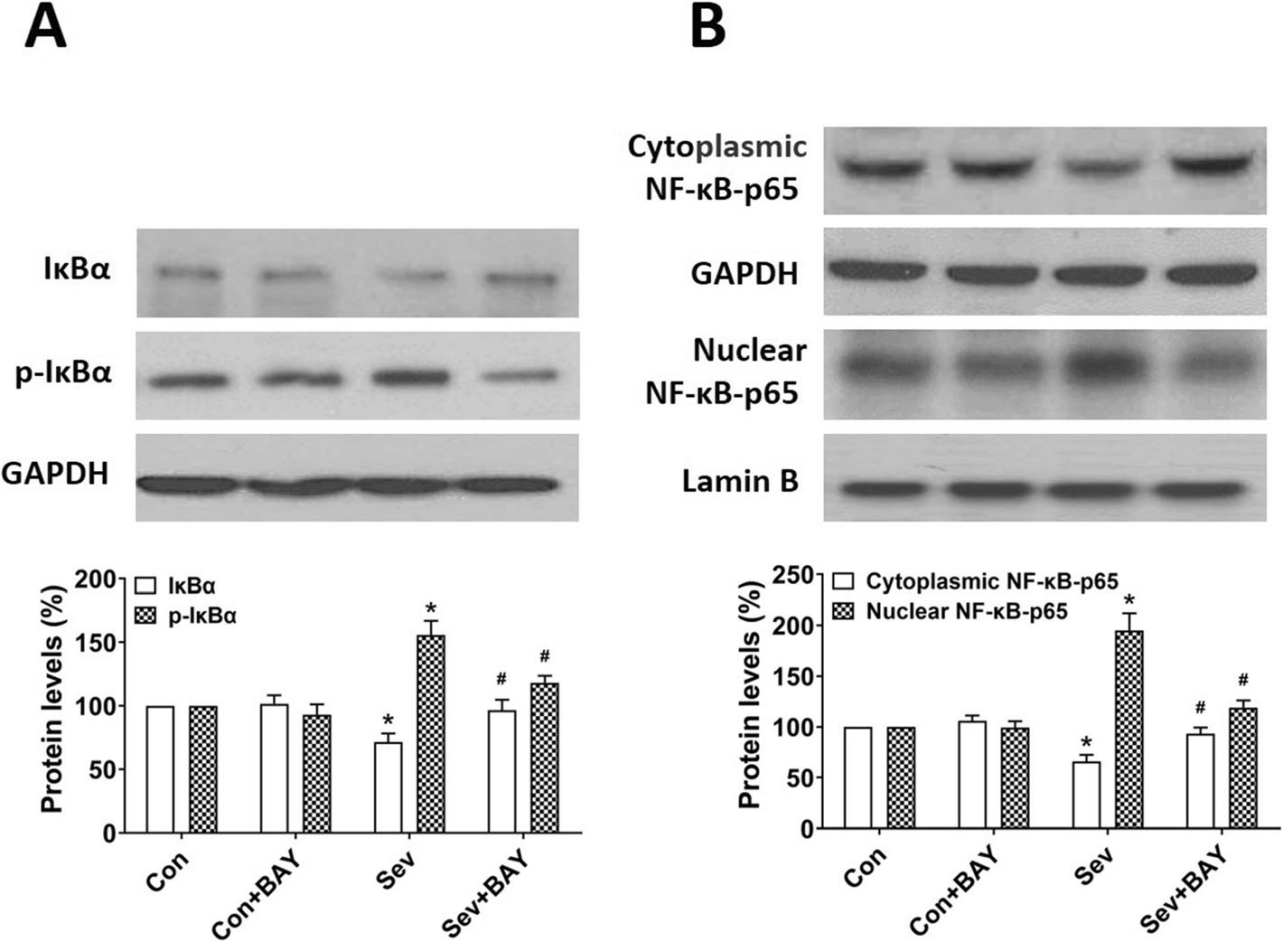
BAY 11-7082 inhibits sevoflurane-induced NF-κB activation. Rat pups at PND 6 were randomly assigned to one of four treatment protocols: control + vehicle (Con group), control + BAY 11-7082 (Con + BAY group), sevoflurane + vehicle (Sev group), and sevoflurane + BAY 11-7082 (Sev + BAY group). BAY 11-7082 (20 mg/kg) or PBS (vehicle) was intraperitoneally administered to the pups 30 min before gas inhalation. Sevoflurane anesthesia was induced by placing the rat pups in an anesthetizing chamber delivering 3% sevoflurane for 2 h daily for three consecutive days. For the control condition, 30% O_2_ was delivered at the same flow rate. **A**, **B** Representative western blotting and quantitative analysis of the protein levels of IκBα, p-IκBα, and cytoplasmic and nuclear NF-κB from fresh hippocampal tissue homogenates obtained at PND 8. Values are presented as mean ± SEM (*n* = 6 rats/group). ^*^p < 0.05 versus the Con group; ^#^*p* < 0.05 versus the Sev group

Sevoflurane has been shown to up-regulate the NF-κB signaling pathway in the hippocampus in neonatal rats [2, 5]. We observed that the level of phosphorylated IκBα protein (p-IκBα) significantly increased, and the total level of IκBα significantly decreased in the developing rat hippocampus after repeated sevoflurane exposure (Fig. 1A). Moreover, the protein level of nuclear NF-κB p65 increased, while the level of cytoplasmic NF-κB p65 decreased in the sevoflurane group, as compared with the control group (Fig. 1B). BAY 11-7082, the most commonly used NF-κB activation inhibitor, can freely cross the blood–brain barrier [24]. We further investigated the effect of BAY 11-7082 on the expression of NF-κB and IκB. Notably, changes in the protein levels of NF-κB p65 and IκBα induced by sevoflurane GA were reversed by the administration of BAY 11-7082 (Fig. 1). Our results indicated that repeated sevoflurane induces activation of NF-κB signaling in the hippocampus in neonatal rats, and this activation is successfully inhibited by BAY 11-7082 administration.

### Inhibition of NF-κB by BAY 11-7082 suppresses the activation of canonical and non-canonical inflammatory caspases in rats with sevoflurane anesthesia

**Fig. 2:**
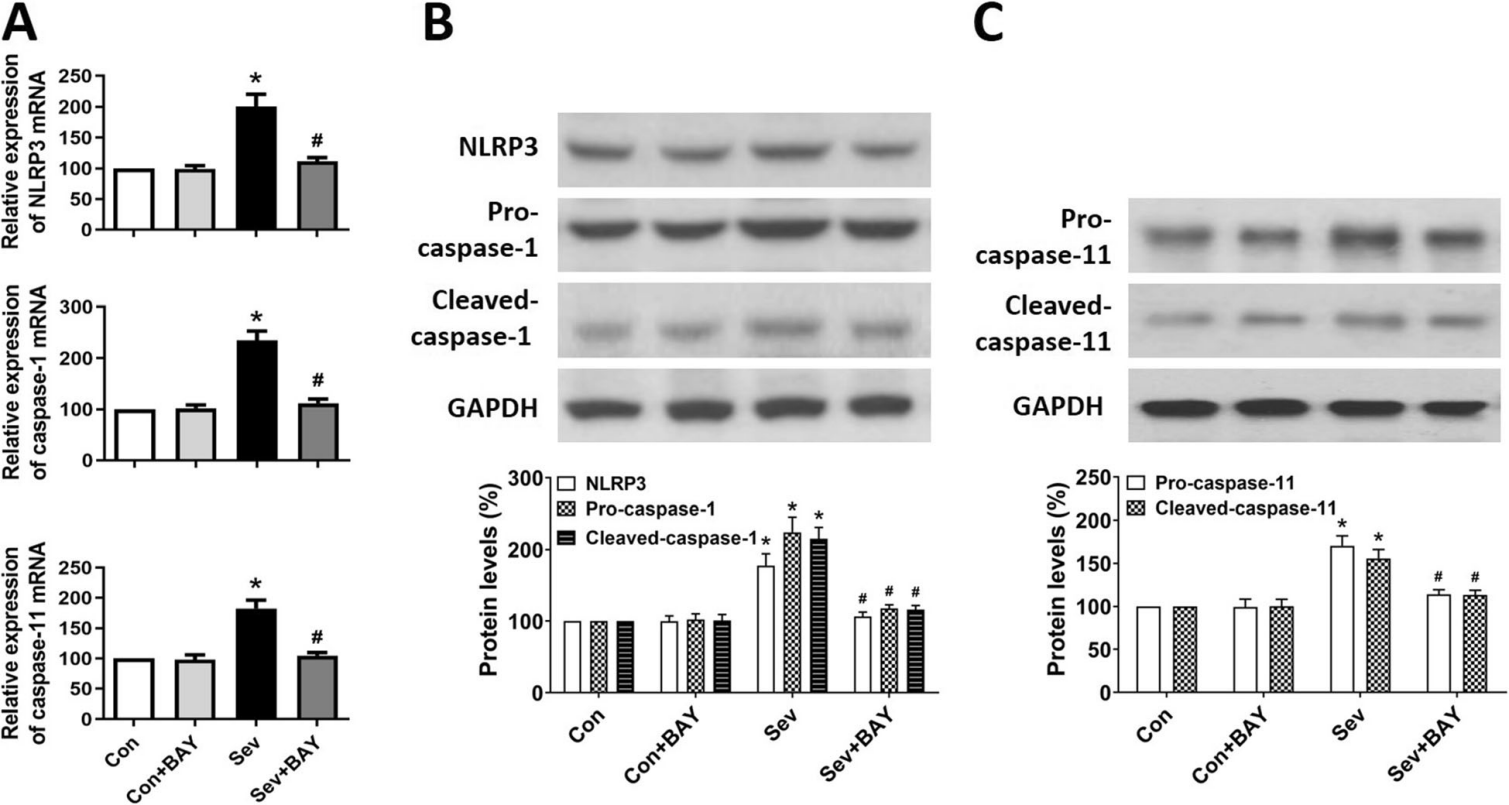
BAY 11-7082 suppresses sevoflurane-induced activation of canonical and non-canonical inflammatory caspases. **A** Representative real-time PCR analysis of the mRNA levels of NLRP3, caspase-1, and caspase-11 in the hippocampus in developing rats. **B** Representative western blotting and quantitative analysis of the protein levels of NLRP3, pro-caspase-1, and cleaved caspase-1 in the hippocampus in developing rats. **C** Representative western blotting and quantitative analysis of the protein levels of pro-caspase-11 and cleaved caspase-11 in the hippocampus in developing rats. Values are presented as mean ± SEM (*n* = 6 rats/group). ^*^p < 0.05 versus the Con group; ^#^*p* < 0.05 versus the Sev group

Pyroptosis can be triggered by the canonical (caspase-1-mediated) and non-canonical (caspase-11-mediated) inflammasome signaling pathways. Both processes are tightly controlled by the activation of NF-κB [13–15]. We observed that the mRNA levels of NLRP3, caspase-1, and caspase-11 clearly increased in the hippocampus in neonatal rats after repeated sevoflurane exposure (Fig. 2A). In addition, the protein levels of NLRP3, pro-caspase-1, cleaved-caspase-1, pro-caspase-11, and cleaved-caspase-11 were significantly higher in the sevoflurane group than the control group (Fig. 2B, C). These changes are molecular characteristics of the canonical and non-canonical pyroptotic pathways. Remarkably, in the rats pre-treated with BAY 11-7082, sevoflurane did not increase either the mRNA or protein levels of NLRP3, caspase-1, and caspase-11 (Fig. 2), thus suggesting that inhibition of NF-κB activation by BAY 11-7082 suppresses both the canonical and non-canonical pyroptotic pathways after sevoflurane anesthesia exposure.

### Inhibition of NF-κB by BAY 11-7082 attenuates sevoflurane-induced pyroptosis and neuroinflammation

**Fig. 3:**
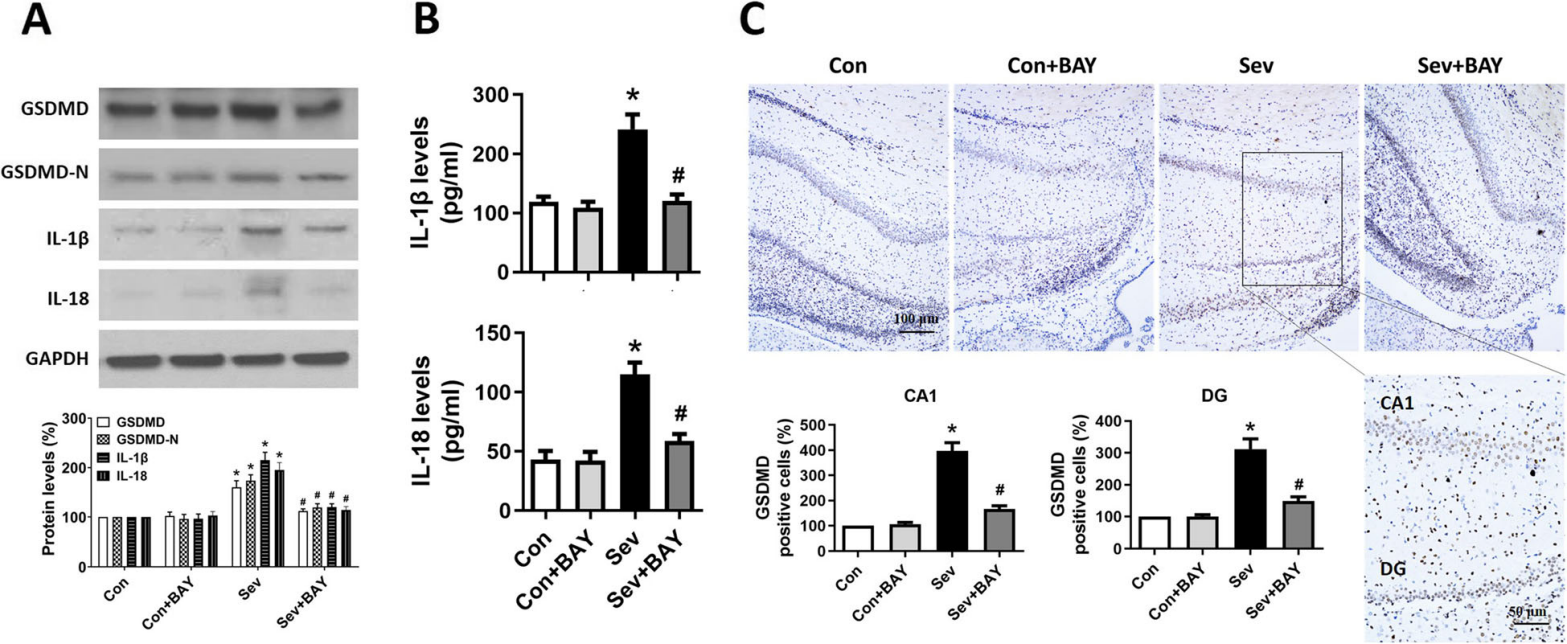
BAY 11-7082 attenuates sevoflurane-induced pyroptosis and neuroinflammation in the rat hippocampus. **A** Representative western blotting and quantitative analysis of protein levels of GSDMD, GSDMD-N, IL-1β, and IL-18 in the hippocampus in developing rats. **B** ELISA analysis of IL-1β and IL-18 levels. **C** Representative images of GSDMD staining in the hippocampal CA1 and DG regions. Scale bar = 100 μm for all photographs except for the last one (scale bar = 50 μm). The lower panel shows the number of GSDMD-positive cells in the CA1 and DG regions of the hippocampus in the four experimental groups. Values are presented as mean ± SEM (*n* = 6 rats/group). ^*^p < 0.05 versus the Con group; ^#^*p* < 0.05 versus the Sev group

**Fig. 4:**
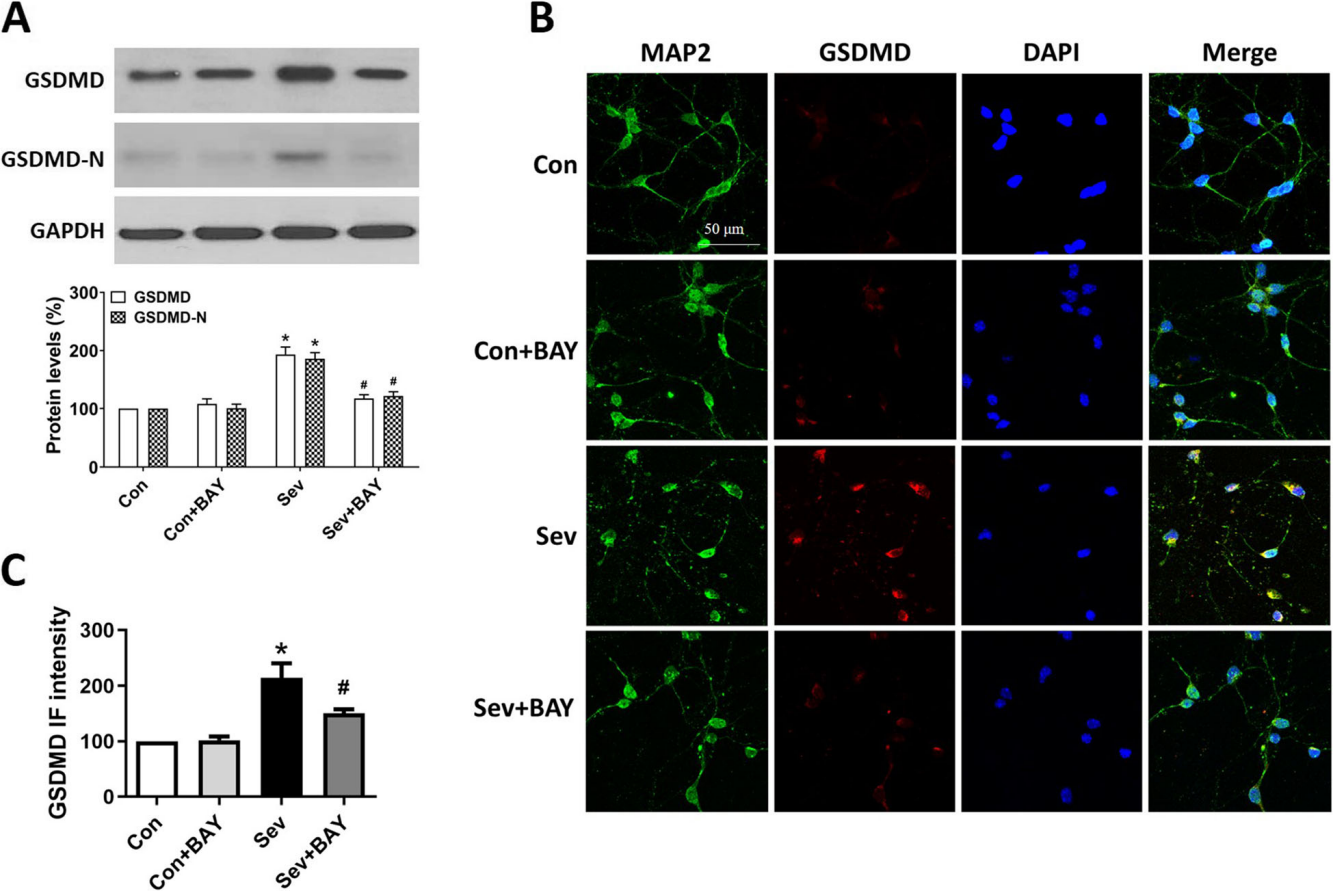
BAY 11-7082 attenuates sevoflurane-induced pyroptosis in primary hippocampal neurons. **A** Representative western blotting and quantitative analysis of the protein levels of GSDMD and GSDMD-N from primary hippocampal neuron homogenates obtained on DIV 11. **B** Representative immunofluorescence staining images of MAP2, GSDMD, and DAPI in primary hippocampal neurons. Scale bars = 50 μm. **C** Quantitative analysis of the intensity of GSDMD-immunofluorescence in primary hippocampal neurons. Values are presented as mean ± SEM (*n* = 6 cultures/group). ^*^p < 0.05 versus the Con group; ^#^*p* < 0.05 versus the Sev group

GSDMD is the substrate of active caspase-1 and caspase-11, and the executor of proptosis. Activated caspase-1 or caspase-11 cleaves the N- and C-terminals of GSDMD and triggers pyroptosis [7–10]. In an in vivo study, we found that repeated sevoflurane exposure induced upregulation of GSDMD, GSDMD-N, and the inflammatory cytokines IL-1β and IL-18 (Fig. 3A, B) in the hippocampus in developing rats. These findings were further confirmed by the increased number of GSDMD-positive cells observed in the CA1 and DG areas of brain sections in the sevoflurane-treatment group (Fig. 3C). In an in vitro study, western blotting analysis from primary neuronal cultures showed that the protein levels of GSDMD and GSDMD-N significantly increased in hippocampal neurons after repeated sevoflurane exposure (Fig. 4A). Moreover, immunocytochemistry staining showed that the GSDMD immunofluorescence intensity was elevated in the sevoflurane group (Fig. 4B, C). Notably, BAY 11-7082 attenuated the cleavage of GSDMD and the release of inflammatory cytokines (Figs. 3 and 4), thus suggesting that inhibition of NF-κB by BAY 11-7082 successfully attenuates the pyroptosis and neuroinflammation induced by sevoflurane anesthesia.

### BAY 11-7082 rescues sevoflurane-induced neuronal damage and synaptic dysfunction

**Fig. 5:**
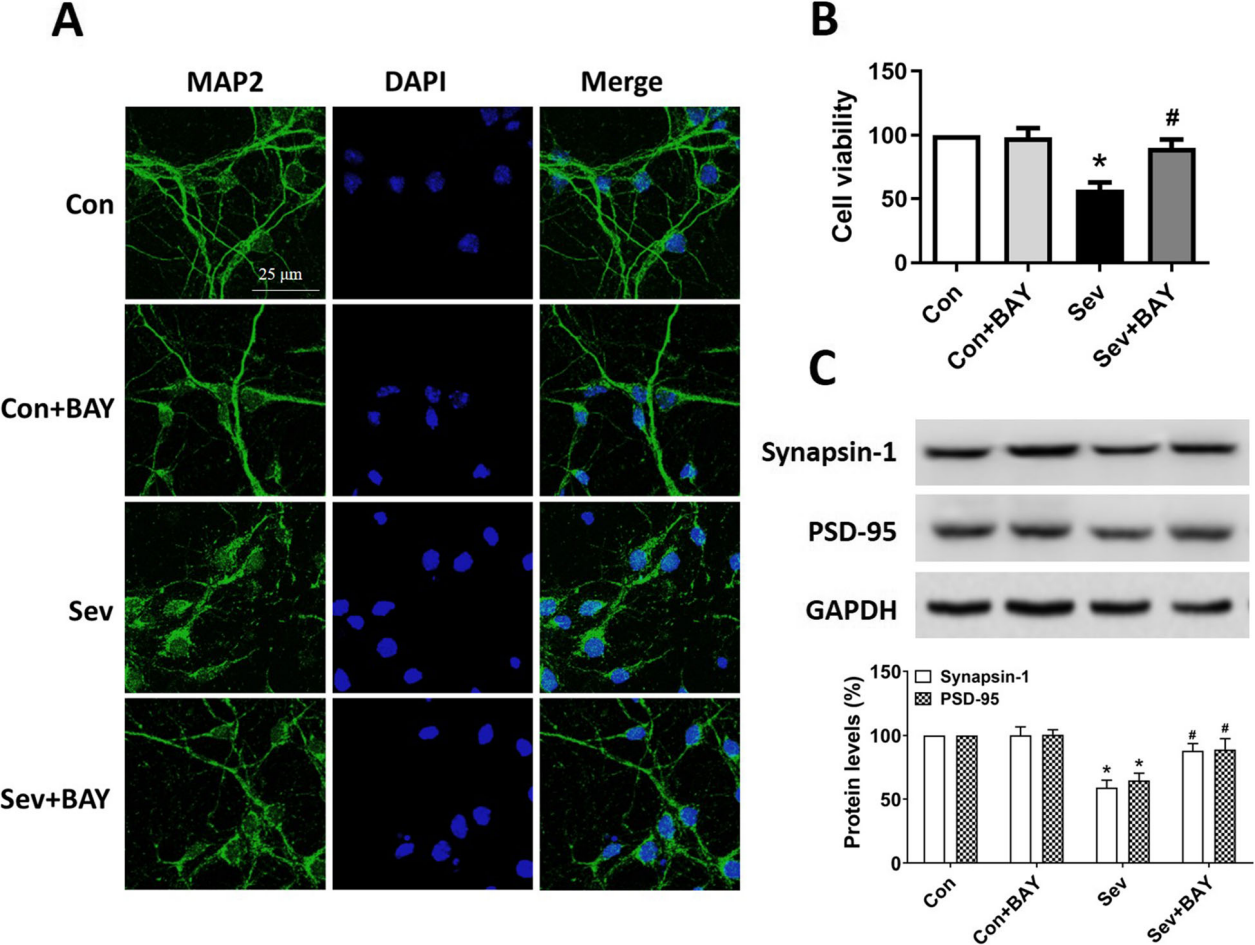
BAY 11-7082 rescues sevoflurane-induced neuronal damage and synaptic dysfunction. **A** Representative immunofluorescence images of MAP2 staining in primary hippocampal neurons. Scale bar = 25 mm for all photographs. **B** Cell viability of cultured hippocampal neurons on DIV 11. **C** Representative western blotting and quantitative analysis of protein levels of Synapsin-1 and PSD-95 in the hippocampus in developing rats. Values are presented as mean ± SEM (*n* = 6 cultures or rats/group). ^*^p < 0.05 versus the Con group; ^#^*p* < 0.05 versus the Sev group

Pyroptosis plays important roles in regulating neuronal cell death, and in maintaining synaptic integrity and thereby modulating neural networks. To test whether attenuating NF-κB-mediated pyroptosis might rescue sevoflurane-induced neuronal damage, we treated primary neurons with BAY 11-7082, then performed MAP2 immunostaining to examine neuronal morphology and outgrowth. Neurons under control condition had extensive neurite branches, processes, and overlapping neurites while those exposed to sevoflurane displayed impaired morphology and dystrophic neurites (Fig. 5A). Remarkably, neurons pre-treated with BAY 11-7082, compared with those in the sevoflurane group, showed significantly improved morphology. The treatment did not alter neuron morphology in the Con + BAY group, thus indicating that NF-κB inhibitors do not generally improve neuronal outgrowth and branching, but instead protect against sevoflurane-mediated neurotoxicity. In addition, cell viability assays showed that BAY 11-7082 treatment increased neuronal viability in the neurons exposed to sevoflurane compared with vehicle alone (Fig. 5B).

We further measured the protein levels of Synapsin-1 and PSD-95, two important indicators of synaptic structure [25], in the hippocampus in developing rats. Repeated sevoflurane exposure induced downregulation of Synapsin-1 and PSD-95, whereas BAY 11-7082 pretreatment significantly attenuated this downregulation (Fig. 5C), thus suggesting that BAY 11-7082 protects synaptic integrity. Collectively, our results provide important evidence that BAY 11-7082 rescues sevoflurane-induced neuronal damage and synaptic dysfunction.

### BAY 11-7082 ameliorates sevoflurane-induced neurocognitive deficits in adolescent rats

**Fig. 6:**
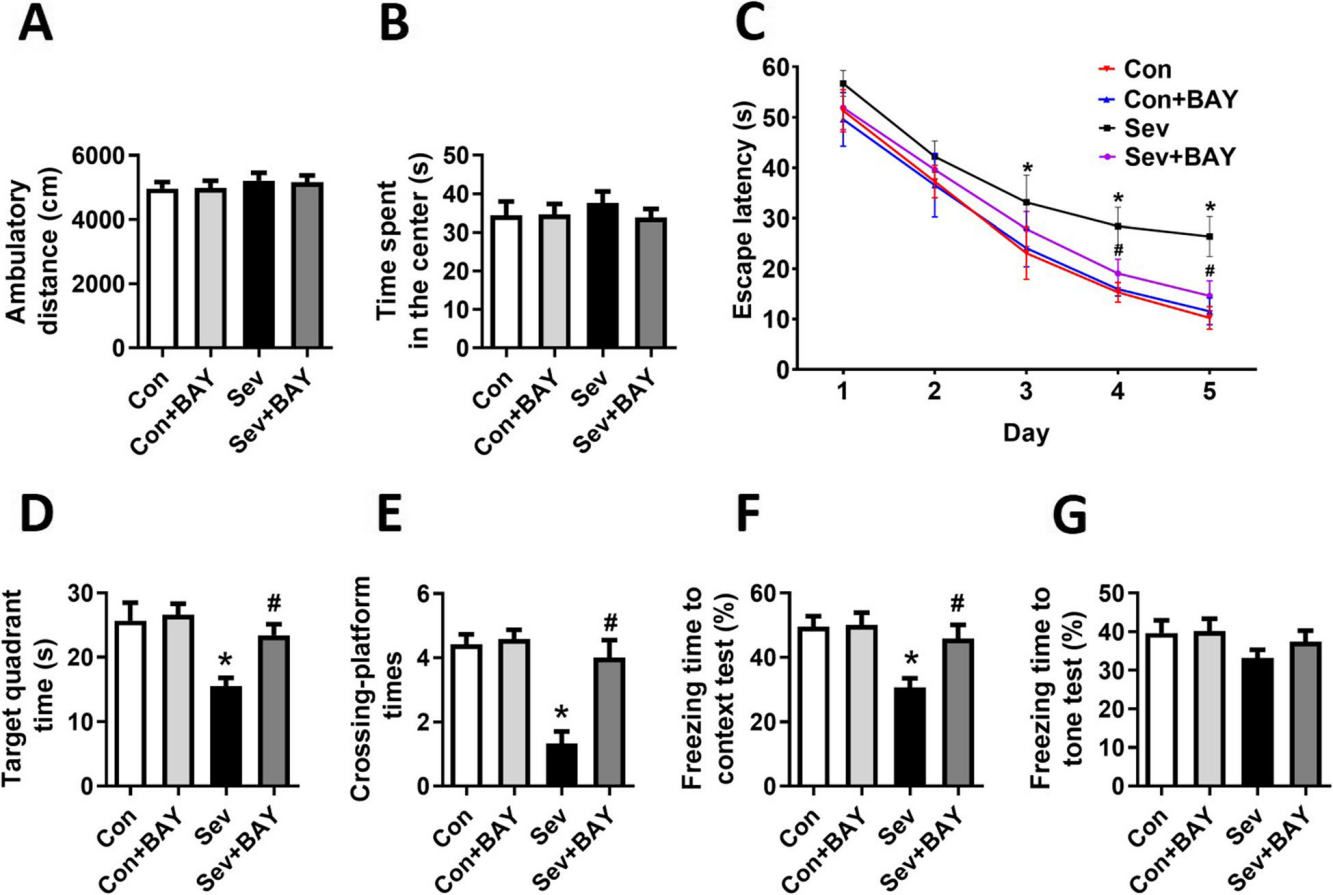
BAY 11-7082 ameliorates sevoflurane-induced cognitive deficits in adolescent rats. **A** Total distance traveled and **B** time spent in the center in open field tests. The open field tests in developing rats were performed at PND 40. **C** Escape latency during the spatial training of MWM for five consecutive days. The MWM tests were performed at PND 50. **D** Time spent in the target quadrant and **E** crossing platform times in the probe trial of MWM. **F** Freezing time to context and **G** freezing time to tone in the fear conditioning tests. The fear conditioning tests were performed at PND 60. Values are presented as mean ± SEM (*n* = 12 rats/group). ^*^p < 0.05 versus the Con group; ^#^*p* < 0.05 versus the Sev group

To further verify the role of NF-κB-mediated pyroptosis in sevoflurane-induced neurocognitive deficits, we performed open field tests, MWM tests, and fear conditioning tests at PND 40, 50, and 60, respectively. The open field tests showed no differences among the four groups in spontaneous locomotor activity, as reflected by the total distance (Fig. 6A) and the time spent in the center (Fig. 6B), thereby excluding the possibility that locomotor activity *per se* affected the results of the MWM tests and fear conditioning tests.

The MWM test is a hippocampus-dependent memory test for assessing spatial learning and memory [26, 27]. BAY 11-7082 pretreatment successfully shortened the escape latency in the training tests (Fig. 6C) and increased the target quadrant time (Fig. 6D) and crossing platform times (Fig. 6E) in probe trial in developing rats exposed to sevoflurane. The contextual fear conditioning test is an additional test to evaluate the ability of hippocampus-dependent memory [28, 29]. We showed that BAY 11-7082 pretreatment ameliorated the sevoflurane-induced decrease in the percentage freezing time (Fig. 6F). However, we observed no difference in the cued fear conditioning test results among the four groups (Fig. 6G). Our results suggested that NF-κB-mediated pyroptosis may be involved in the pathogenesis of sevoflurane-induced neurocognitive deficits and that BAY 11-7082 has a cognitive protective effect in adolescent rats after early exposure to sevoflurane.

## Discussion

In the present study, we assessed whether NF-κB-mediated pyroptosis might be involved in the pathophysiology of neuroinflammation and cognitive deficits after repeated neonatal sevoflurane exposure in developing rats. We found that repeated neonatal sevoflurane exposure upregulated the expression of NF-κB, NLRP3, caspase-1, and caspase-11, and induced neuroinflammation and pyroptosis in the hippocampus in developing rats. Remarkably, pretreatment with BAY 11-7082, a selective NF-κB inhibitor, inhibited the activation of NF-κB signaling and both canonical and non-canonical pyroptotic pathways. Consequently, BAY 11-7082 rescued the hippocampal neuronal damage and synaptic dysfunction and improved the long-term cognitive function in adolescent rats after early exposure to sevoflurane. Collectively, our findings suggested that NF-κB-mediated pyroptosis may play an important role in sevoflurane-induced cognitive deficits in the developing brain, and NF-κB inhibition might be a potential target for ameliorating neuroinflammation and GA-induced development-related neurocognitive dysfunction.

GA can cause developmental neuronal damage, and perturb synaptogenesis and synapse formation in the developing brain, thus leading to long-term neurocognitive impairment [30–33]. We observed that adolescent rats had poorer learning and memory function after sevoflurane anesthesia. In addition, repeated sevoflurane exposure damaged neuronal morphology and neurite growth in primary neuronal cultures, and induced the downregulation of the synapse-associated proteins Synapsin-1 and PSD-95 in the hippocampus. These findings confirmed previous evidence of the histopathologic changes induced by GA in the developing brain [30–33]. Limited clinical evidence from structural neuroimaging studies has indicated the possibility of brain atrophy in children exposed to anesthesia and surgery [34]. Such brain structural abnormalities might be associated with the histopathologic changes in early anesthesia observed in animal studies, including damaged neuronal morphology and synaptic integrity. These possibilities must be further investigated through neuroimaging.

Neuroinflammation is an essential process in the pathophysiology of GA-induced neuronal damage and cognitive deficits in the developing brain [1–5]. Pyroptosis is a novel and unique inflammatory form of programmed cell death, and GSDMD has recently been identified as the key effector of pyroptosis. Cleavage of GSDMD frees the GSDMD-N domain, which oligomerizes and forms pores on the cell membrane. These pores increase the membrane permeability and facilitate the release of inflammatory cytokines, which subsequently trigger an inflammatory response cascade [7–11]. Pyroptosis has been implicated in the occurrence and development of many inflammatory and non-inflammatory diseases, including sepsis, neurodegenerative diseases, cancer, viral infectious diseases, and cerebral ischemia [7–11]. We and others have recently demonstrated that the GSDMD-induced pyroptosis is involved in cognitive impairment induced by the volatile anesthetic isoflurane in aged mice [12] and in hippocampal neurotoxicity induced by the intravenous anesthetic ketamine in mouse primary hippocampal neurons [35]. Here, our in vitro findings showed that repeated neonatal sevoflurane exposure upregulated the expression of GSDMD and GSDMD-N in primary hippocampal neurons, impaired the neuronal morphology and network, and decreased neuronal viability. In addition, our in vivo studies showed that sevoflurane upregulated the expression of GSDMD, GSDMD-N, IL-1β, IL-18, and synaptic Synapsin-1 and PSD-95 in the hippocampus in developing rats. More importantly, sevoflurane caused long-term cognitive deficits in adolescent rats. Thus, we speculate that GSDMD-induced pyroptosis may be involved in neuroinflammation and neurocognitive impairment after repeated neonatal sevoflurane exposure in the developing brain.

GSDMD is the substrate for active caspase-1 and caspase-11. The canonical caspase-1 pathway can be triggered by the NLRP3 inflammasome, which functions through interaction with apoptosis-associated speck-like protein and the subsequent recruitment of the precursor form of caspase-1 (pro-caspase-1), thus leading to the cleavage of caspase-1 and the maturation of IL-1β and IL-18. The non-canonical caspase-11 inflammasome pathway is activated by lipopolysaccharides in the cytoplasm in infected cells. Upon activation, cleaved caspase1/11 directly mediates GSDMD cleavage and thus serves as an important checkpoint in GSDMD-mediated pyroptosis [7–11]. The levels of pyroptosis-related proteins, including NLRP3, pro-caspase-1, pro-caspase-11, cleaved-caspase-1, and cleaved-caspase-11, significantly increase after multiple doses of ketamine administration in mouse primary hippocampal neurons [35]. Our previous study has also shown that the protein levels of NLRP3 and cleaved-caspase-1 increase in the hippocampus in aged mice after isoflurane anesthesia [12]. In line with these findings, our present data showed that the protein levels of NLRP3, pro-caspase-1, cleaved-caspase-1, pro-caspase-11, and cleaved-caspase-11 were significantly elevated in the hippocampus in neonatal rats after repeated sevoflurane exposure. Consistent results were also found through RT-PCR assays showing similar increases at the mRNA level. These results indicated that both the canonical and non-canonical pyroptotic pathways are activated in the developing brain after sevoflurane anesthesia.

The mechanisms underlying inflammatory caspases activation in pyroptosis are multifaceted. Recent studies have shown that NF-κB is an essential transcription factor in pyroptosis. NF-κB is normally sequestered in the cytoplasm, bound to the regulatory protein IκB. Upon activation, IκB is phosphorylated by the enzyme IκB kinase, thus resulting in the release of NF-κB. The liberated NF-κB then translocates to the nucleus and induces the expression of target genes [36, 37], including *NLRP3*, *caspase-1*, and *caspase-11*, which are involved in canonical and non-canonical inflammatory responses [13–15]. NF-κB signaling, a key factor in the regulation of neuroinflammation, has been reported to be activated by inhalation anesthetics, such as isoflurane and sevoflurane [2, 5, 38, 39]. In this study, repeated sevoflurane exposure decreased IκBα and cytosolic NF-κB p65, and increased p-IκBα and nuclear NF-κB p65 in the hippocampus, thus suggesting that activation of NF-κB signaling occurs after sevoflurane anesthesia. Furthermore, after NF-κB activation, NF-κB translocates into the nucleus, where it initiates the transcription and translation of NLRP3, caspase-1 and caspase-11. Therefore, our findings suggest a correlation between NF-κB activation and pyroptosis in the developing brain after neonatal general anesthetics exposure.

To further demonstrate the role of NF-κB-mediated pyroptosis in cognitive impairment induced by sevoflurane anesthesia, we used BAY 11-7082, a selective inhibitor of NF-κB that irreversibly inhibits IκBα phosphorylation and NF-κB activation [16, 17] and has been found to attenuate inflammation-induced memory injury in neurodegenerative diseases [40, 41]. We found that BAY 11-7082 pretreatment decreased sevoflurane-induced pyroptosis and neuroinflammation by inhibiting the activation of canonical and non-canonical inflammatory caspases. Moreover, BAY 11-7082 protected against neuronal damage and synaptic dysfunction, and attenuated cognitive impairment in developing rats after sevoflurane anesthesia. GSDMD is the executor of pyroptosis, and recent efforts have focused on developing inhibitors to interfere with the pore-forming function of GSDMD. In addition to inhibiting NF-κB activation, BAY 11-7082 potently inhibits GSDMD pore formation in liposomes, as well as inflammasome-mediated pyroptosis and IL-1β secretion in human and mouse cells [42, 43]. Thus, inhibition of GSDMD pore formation appears to be another protective effect of BAY 11-7082 against GA-induced cognitive deficits. Future studies are needed to confirm this hypothesis.

## Conclusion

This study provides the first demonstration that NF-κB-mediated pyroptosis may be involved in neuroinflammation and cognitive impairment after repeated neonatal sevoflurane administration. When the activation of NF-κB is inhibited by BAY 11-7082, the pyroptosis mediated by canonical and non-canonical inflammatory caspases is significantly alleviated. Our study provides a promising strategy for treating cognitive deficits in the developing brain through modulating pyroptosis.

## Data Availability

All data generated in this study are included in this manuscript.

## Abbreviations

GA: General anesthesia
NF-κB: Nuclear factor-κB
TNF-α: Tumor necrosis factor-α
IL-1β: Interleukin-1β
NLRP3: Nod-like receptor pyrin domain-containing 3
GSDMD: Gasdermin D
PND: Postnatal day
DIV: Days in vitro
MAP 2: Microtubule-associated protein-2
MWM: Morris water maze
